# BNC Protects H9c2 Cardiomyoblasts from H_**2**_O_**2**_-Induced Oxidative Injury through ERK1/2 Signaling Pathway

**DOI:** 10.1155/2013/802784

**Published:** 2013-10-10

**Authors:** Fangbo Zhang, Bin Huang, Ye Zhao, Shihuan Tang, Haiyu Xu, Lan Wang, Rixin Liang, Hongjun Yang

**Affiliations:** Institute of Chinese Materia Medica, China Academy of Chinese Medical Sciences, Beijing 100700, China

## Abstract

Buchang naoxintong capsule (BNC) is a traditional Chinese medicine approved for the treatment of cerebrovascular and cardiovascular diseases. However, little is known about the specific protective function or mechanism by which BNC protects against myocardial injury. This research was designed to investigate the cardioprotective effects of BNC in vitro model of hydrogen peroxide (H_2_O_2_)-induced H9c2 rat cardiomyoblasts. BNC intestinal absorption liquid was used in this study instead of drug-containing serum or extracting solution. Our study revealed that BNC preconditioning enhanced antioxidant function by increasing the activities of total-antioxygen capacity, total-superoxide dismutase, and catalase and by decreasing the production of reactive oxygen species and malondialdehyde. BNC preconditioning also activated extracellular signal-regulated kinases (ERK1/2) and inhibited apoptosis-related proteins such as poly ADP-ribose polymerase (PARP) and caspase-3. Additionally, preincubation with BNC reduced intracellular Ca^2+^ concentration, improved mitochondrial membrane potential, and decreased the apoptosis rate of H9c2 cells in a dose-dependent manner. These data demonstrated that BNC protects H9c2 cardiomyoblasts from H_2_O_2_-induced oxidative injury by increasing antioxidant abilities, activating ERK1/2, and blocking Ca^2+^-dependent and mitochondria-mediated apoptosis. Based on our results, the potency of BNC for protecting H9c2 cells from oxidative damage is comparable to that of trimetazidine.

## 1. Introduction

Oxidative stress plays a critical role in the pathophysiology of several major cardiovascular diseases such as atherosclerosis, hypertension, heart failure, and myocardial ischemical reperfusion injury [1, 2]. Significant oxidative stress causes excessive production of reactive oxygen species (ROS), which is an important event in the development of cardiovascular diseases. ROS accumulation may contribute to a number of cardiovascular disorders [3, 4]. Cellular sources of ROS come from the mitochondrial electron transport chain, xanthine oxidase, NADPH oxidase, lipoxygenase/cyclooxygenase, nitric oxide synthase, and autoxidation of various substances particularly catecholamines [5].

Increased ROS cause significant damage to myocardial cells, which can be neutralized by antioxidant molecules such as superoxide dismutase (SOD), catalase (CAT), and glutathione (GSH). These antioxidant molecules counteract oxidation and play vital roles in maintaining a stable intracellular environment. Moreover, ROS damage cellular membrane by causing lipid peroxidation. Malondialdehyde (MDA) is a major lipid peroxidation product and may reflect the degree of cellular injury [6]. 

Extracellular signal-regulated kinases (ERKs) have been reported to be activated by oxidative stress in some cell types and play important roles in many aspects of cellular function [7]. ERK1/2 activation by ROS in cardiomyocytes mediates a wide range of activities including metabolism, migration, inflammation, cell survival, and cell death [8]. Indeed, DNA microarray data showed that nearly 100 genes were involved in cellular responses to oxidative stress [9].

Buchang naoxintong capsule (BNC) consists of 16 various kinds of traditional Chinese medicines including* Astragalus membranaceus, Salvia miltiorrhiza*, *Ligusticum*, *Radix paeoniae rubra*, *Szechwan lovage Rhizome*, *Semen persicae*, *Carthamus tinctorius L*,* Frankincense*,* myrrh*,* Spatholobus suberectus*, *Achyranthes Root*, *Cassia Twig*, *Mulberry Twig*, earthworms, scorpions, and hirudo, which is an approved traditional Chinese medicine for stroke and angina [10]. BNC protected mice from atherosclerosis by reducing lipid concentrations and inhibiting maturation of dendritic cells [11]. BNC also increased the catalytic activity of the drug-metabolizing CYP2C19 enzyme [12]. The combination of BNC and clopidogrel enhanced the antiplatelet effect in patients with the *CYP2C19∗*2 gene mutation [13]. BNC has been shown to have protective effects in cardiac and vascular diseases. However, the cellular and molecular mechanisms of BNC cardioprotective activity have not yet been elucidated.

In this study, oxidative stress was induced by exposing H9c2 cells to H_2_O_2_, which is a well-established model [14]. Treatments consisted BNC intestinal absorption liquids rather than extraction solutions or drug-containing sera. Trimetazidine (TMZ), a common treatment for angina in cardiac patients, served as a control [15, 16].

## 2. Materials and Methods

### 2.1. Animals

Adult male Sprague Dawley rats weighing 220 ± 10 grams (g) were purchased from the Experimental Animal Center of Peking University Health Science Center, Beijing, China (certificate no. SCXK (Jing) 2009-0017). The experiment was approved by the local Committee on Animal Care and Use.

### 2.2. Materials

3-(4,5-Dimethylthiazol-2-yl)-2,5-diphenyltetrazolium bromide (MTT), TMZ, and H_2_O_2_ solution (35 wt.% in water) were purchased from Sigma (USA). The JC-1 commercial kit was obtained from Beyotime (China). The 2′,7′-dichlorofluorescein diacetate (DCFH-DA) probe, the total-antioxygen capacity (T-AOC) kit, the total superoxide dismutase (T-SOD) kit, the CAT kit, and the MDA kit were obtained from Nanjing Jiancheng (China). BNCs (Med-drug Permit no. Z20025001) were obtained from Buchang Pharmaceutical Co. Ltd. Pharmaceutical standard materials for the liquid chromatography experiment were purchased from the National Institutes for Food and Drug Control in China. All chemicals were of analytical grade.

### 2.3. Preparation of BNC Intestinal Absorption Liquid and Treatment of Rat Intestines

The powder from 100 g BNC was dissolved in 2,000 mL 95% ethanol. This solution was heated until boiling for 2 hours (h) under reflux and then filtered. Water was added in the filtrate to make a 200-mL BNC extraction solution. The 200 mL BNC extraction solution was evaporated with a rotary evaporator until almost completely dry. Tyrode's buffer solution (NaCl 8.00 g, KCl 0.28 g, NaHCO_3_ 1.00 g, NaH_2_PO_4_ 0.05 g, MgCl_2_ 0.10 g, CaCl_2_ 0.20 g, glucose 1.00 g, pH 7.4) was added to bring the volume of BNC solution to 600 mL. Rats were maintained in fasting conditions for 12 h before the experiment. Anesthesia was provided, and the intestine of each rat was quickly removed. Intestines were washed with Tyrode buffer solution (0°C) and cut into four 14 cm segments. Each segment was turned inside-out and ligated to form a sac at one end. The sac filled with Tyrode buffer was incubated in Magnus' bath for 5 minutes (min) to reach equilibration. Buffer was then exchanged with BNC solution (25 mL), which was maintained at 37°C and continuously injected with O_2_/CO_2_ (95%/5%). Serosal-side solutions containing absorbed constituents (2 mL) were drained into tubes after 2 h. BNC intestinal absorption liquids were filtered with a microfiltrate membrane (0.22 *μ*m) and stored at −20°C. The original concentration of BNC intestinal absorption liquid was 1 mg/mL (crude drug).

### 2.4. Liquid Chromatography

BNC intestinal absorption liquid was analyzed by Ultra Performance Liquid Chromatography (UPLC) with a Waters ACQUITY C18 column (2.1 mm × 150 mm, 1.7 *μ*m). The mobile phase consisted of acetonitrile and 0.5% formic acid with gradient elution at a flow rate of 0.3 mL/min. The detection wavelengths were set at 235, 280, 324, and 400 nm. The injection volume was 2 *μ*L and the column temperature was maintained at 30°C. 

### 2.5. Cell Culture and Treatment

The H9c2 cell line was obtained from the Cell Resource Center of Peking Union Medical College in China. Cells were cultured in high-glucose DMEM (Invitrogen, USA) supplemented with 10% fetal calf serum (Hangzhou Sijiqing, China) and a combination of penicillin-streptomycin in a humidified 5% CO_2_ atmosphere at 37°C. Cells were nearly 80% confluent and then treated with different concentrations of BNC for 24 h before addition of 100 *μ*M H_2_O_2_ for 1 h.

### 2.6. MTT Assay

The optimum concentration and application time for H_2_O_2_ and the protective effect of BNC on H9c2 cells were determined by MTT assay. Cells were dispersed by trypsinization and seeded at (8,000–10,000) cells/well in a 96-well plate overnight before being treated. Subsequently, 20 *μ*L MTT solution (5 mg/mL) was added to each well and incubated at 37°C for 4 h. The supernatant was removed, and the insoluble formazan product was dissolved in 150 *μ*L DMSO. Absorbance of each culture well was measured with a microplate reader (Molecular Devices, USA) at a wavelength of 570 nm. 

### 2.7. Biochemical Analysis of H9c2 Cell Lysate

H9c2 cells were adjusted to 1 × 10^6^ cell/mL after trypsinization, washed with phosphate-buffered saline (PBS) twice, and centrifugated at 1,500 rpm/min for 10 min. The supernatant were then removed. The precipitate obtained through centrifugation was crushed by ultrasonic wave, and the cell lysates were resuspended. T-AOC, T-SOD, CAT, and MDA were determined with a microplate reader (Molecular Devices, USA) according to the protocol of the detection kit. Protein content was measured with the BCA Bradford protein assay (Pierce, USA).

### 2.8. Detection of Intracellular ROS

Intracellular ROS was measured with the DCFH-DA probe. Digestive H9c2 cell suspensions were incubated with 10 *μ*M DCFH-DA for 30 min and then washed three times with PBS to remove residual probe. The cellular fluorescence intensities were measured with a fluorescence microplate reader (Molecular Devices, USA). Excitation and emission wavelengths were set at 488 nm and 525 nm, respectively.

### 2.9. Detection of Apoptosis with Annexin V-PI

H9c2 cells were harvested with 0.25% trypsin, washed twice with cold PBS (4°C), and resuspended in 500 *μ*L binding buffer. Cells were incubated with 10 *μ*L Annexin V for 60 min in the dark at 4°C, and then with 5 *μ*L propidium iodide for 5 min at room temperature. Fluorescence was analyzed with a FACStar Plus flow cytometer (Becton-Dickinson, USA).

### 2.10. Measurement of Cytosolic Ca^2+^ Concentration

Cytosolic Ca^2+^ was assessed with the cell-permeable and high-affinity fluorescent Ca^2+^ indicator Fluo-3/AM. H9c2 cells were collected with trypsin and centrifuged (1,000 rpm, 5 min). Cells were washed with PBS and incubated with 5 mM Fluo-3/AM dye for 40 min at 37°C. The concentration of Ca^2+^ was measured with a FACStar Plus flow cytometer (Becton-Dickinson, USA).

### 2.11. Measurement of Mitochondrial Membrane Potential (MMP)

H9c2 cells were harvested with trypsin and centrifuged (1000 rpm, 5 min). Medium (1 mL) and 5 *μ*M JC-1 dye (1 mL) were added to the centrifugal precipitation for 20 min at 37°C. Cells were washed with dye buffer twice and detected with a FACStar Plus flow cytometer (Becton-Dickinson, USA). Mean FL2 fluorescence intensity indicated the mitochondrial membrane potential.

### 2.12. Western Blot Analysis

Protein samples were prepared from H9c2 cells with RIPA buffer (Beijing Baosai, China). Protein content was measured with the BCA Bradford protein assay. Cell lysates were separated by 10% SDS-PAGE and transferred onto PVDF membranes (Millipore, USA). Membranes were incubated with primary antibody (Cell Signaling Technology, USA) and then with HRP-conjugated secondary antibody (Zhongshanjinqiao, China). Proteins were detected with an enhanced chemiluminescence agent (Millipore, USA). *β*-actin (Cell Signaling Technology, USA) served as an internal control.

The antibodies included rabbit monoclonal antibodies against caspase-3 (1 : 1000), PARP (1 : 1000), ERK1/2 (1 : 1000), p-ERK1/2 (1 : 1000), *β*-actin (1 : 1000), and horseradish peroxidase-conjugated secondary antibodies (goat anti-rabbit, 1 : 2000). 

### 2.13. Statistical Analysis

The experiment was performed at least 3 times, and all values are expressed as means ± standard deviation (SD). Data were compared by one-way ANOVA. Values of *P* < 0.05 were considered statistically significant.

## 3. Results

### 3.1. Qualitative Analysis of BNC Intestinal Absorption Liquid

**Figure 1:**
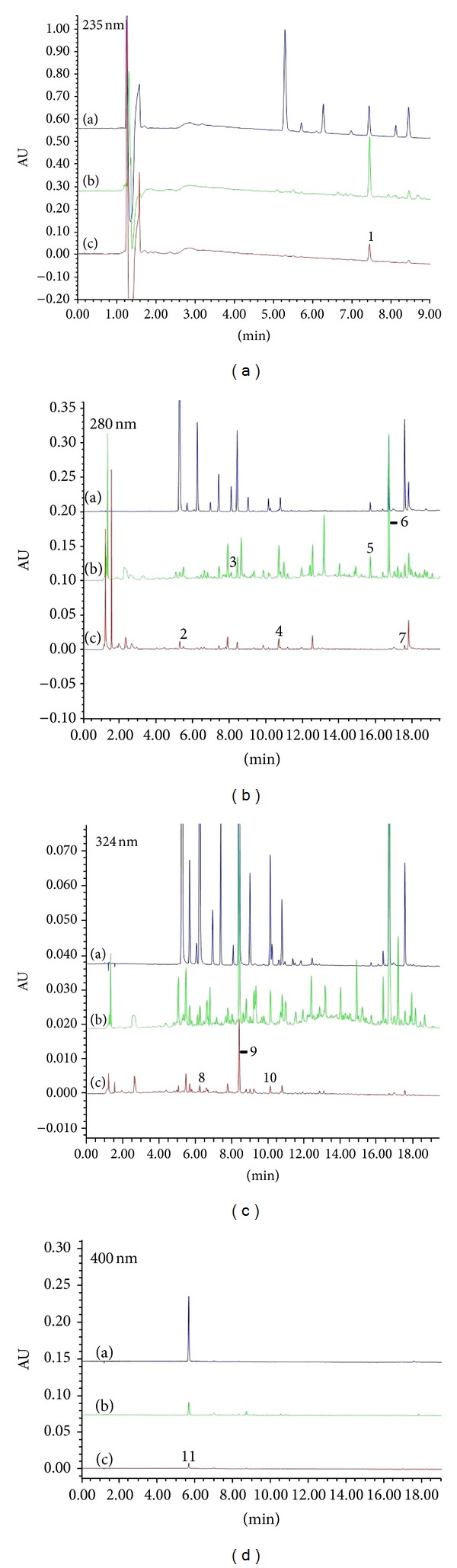
UPLC of Control (a), BNC extraction solution (b), and BNC intestinal absorption liquid (c). Chromatograms numbered from 1 to 11 represent paeoniflorin, protocatechualdehyde, calycosin, salvianolic acid B, senkyunolide A, ligustilide, tanshinone I, caffeic acid, ferulic acid, rosmarinic acid, and hydroxysafflor yellow A.

Eleven constituents were detected in BNC extraction solution. These were calycosin, ligustilide, paeoniflorin, protocatechualdehyde, salvianolic acid B, senkyunolide A, tanshinone I, caffeic acid, ferulic acid, rosmarinic acid, and hydroxysafflor yellow A. Eight constituents in BNC intestinal absorption liquid were examined, which is consistent with BNC extraction solution except calycosin, ligustilide, and senkyunolide A. Results indicated that the composition of BNC intestinal absorption liquid was similar to that of BNC extraction solution (Figure 1).

### 3.2. Working Concentration and Incubation Time of H_**2**_O_**2**_

**Figure 2:**
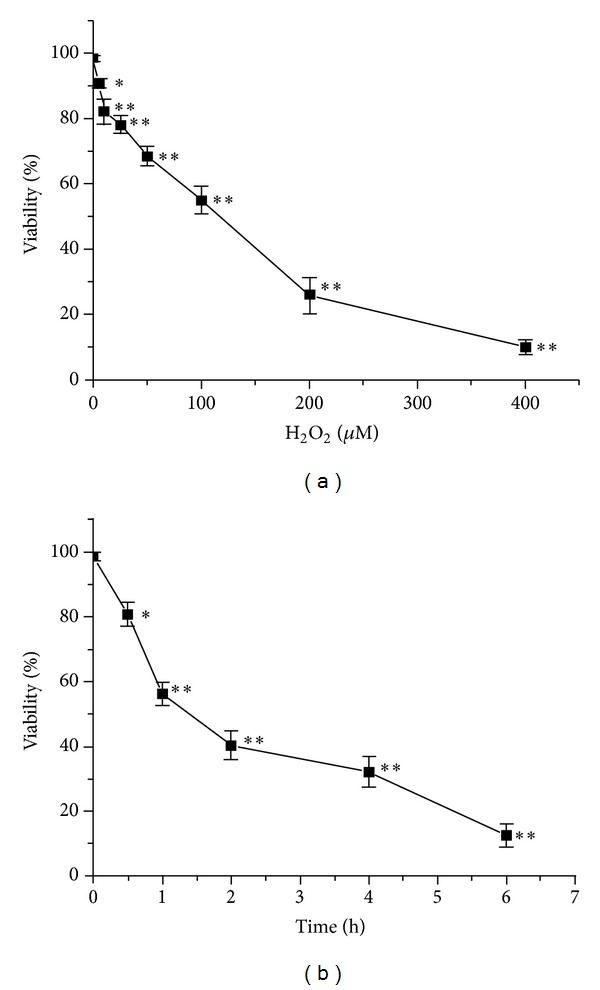
Determination of H_2_O_2_ working conditions. H9c2 cells were cultured with different concentrations of H_2_O_2_ for 1 h (a) or with 100 *μ*M for different incubation times (b). Cell viability was detected by MTT assay. Values are expressed as mean ± SD from three independent experiments. **P < 0.01 versus control; *P < 0.05 versus control.

Treatment with increasing concentrations of H_2_O_2_ (0, 5, 10, 25, 50, 100, 200, or 400 *μ*M) for 1 h caused dose-dependent loss of cell viability. Incubation with 100 *μ*M H_2_O_2_ at different time points (0, 0.5, 1, 2, 4, or 6 h) showed time-dependent reductions in viability. Treatment with 100 *μ*M H_2_O_2_ for 1 h reduced cell viability, and survival rates were 57.87 ± 4.25% (Figure 2(a)) and 52.37 ± 3.90% (Figure 2(b)). Thus, cells were treated with this concentration in subsequent experiments. 

### 3.3. Effect of H_**2**_O_**2**_ on H9c2 Cell Viability

**Figure 3:**
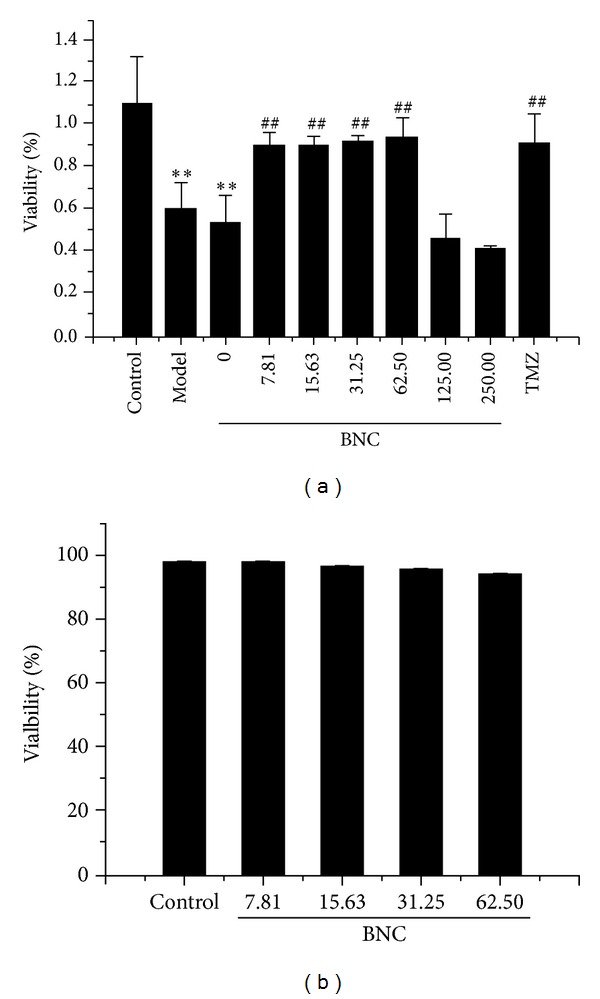
BNC rescued H_2_O_2_-induced loss of cell viability. H9c2 cells were pretreated with different concentrations of BNC (0, 7.81, 15.63, 31.25, 62.50, 125, or 250 *μ*g/mL) for 24 h and then treated with 100 *μ*M H_2_O_2_ for 1 h (a). H9c2 cells were preconditioned with different concentrations of BNC (7.81, 15.63, 31.25, or 62.50 *μ*g/mL) for 24 h (b). Cell viability was measured by MTT assay. Values are expressed as mean ± SD from three independent experiments. **P < 0.01 versus control; ^##^ *P* < 0.01 versus model.

H9c2 cells were treated with H_2_O_2_ (100 *μ*M) for 1 h in the absence or presence of BNC (0, 7.81, 15.63, 31.25, 62.50, 125, or 250 *μ*g/mL). TMZ (10 *μ*M) served as the control drug. Cell viability was measured by MTT assay (Figure 3(a)). Treatment with H_2_O_2_ significantly decreased the viability of H9c2 cells compared to control treatment. Pretreatment with 7.81, 15.63, 31.25, or 62.50 *μ*g/mL BNC for 24 h increased cell viability, demonstrating a dose-dependent response. The results showed that 7.81, 15.63, 31.25, and 62.50 *μ*g/mL BNC protected H9c2 cells from oxidative damage. H9c2 cells were incubated with 7.81, 15.63, 31.25, and 62.50 *μ*g/mL BNC for 24 h, and none of these BNC solutions caused damage to H9c2 cells (Figure 3(b)).

### 3.4. Morphologic Changes in H9c2 Cells

**Figure 4:**
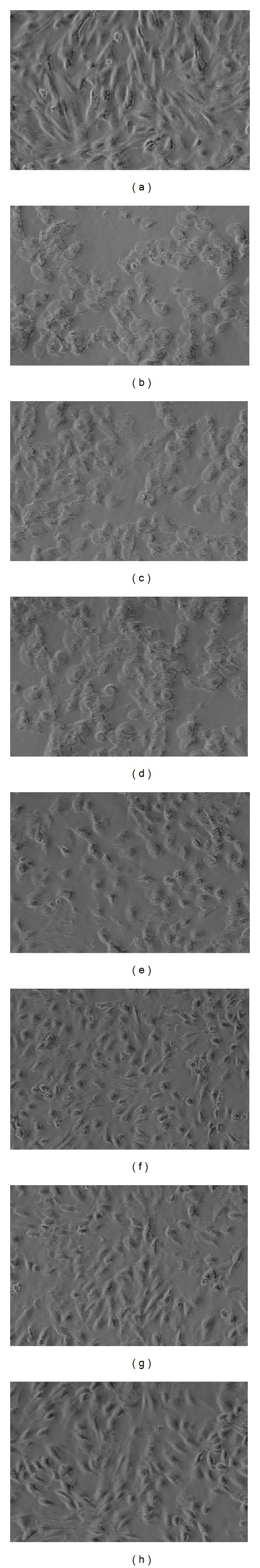
Changes in H9c2 cell morphology in response to H_2_O_2_ and/or BNC. (a) H9c2 cells without BNC or H_2_O_2_ (control); (b) H9c2 cells exposed to 100 *μ*M H_2_O_2_ (model); ((c)–(h)) H9c2 cells pretreated with 0, 7.81, 15.63, 31.25, 62.50 *μ*g/mL BNC or with 10 *μ*M TMZ followed by treatment with 100 *μ*M H_2_O_2_. Magnification: 200x.

The morphologies of H9c2 cells treated with H_2_O_2_ (100 *μ*M) in the presence of BNC (7.81, 15.63, 31.25, or 62.50 *μ*g/mL) were observed with an inverted phase-contrast microscope (Olympus, Japan). Control H9c2 cells were normal with long fusiform shapes. H_2_O_2_ treatment induced distinctive morphological changes, such as cell shrinkage, irregular shape, and a wider intercellular gap. However, the proportion of abnormal cells in 15.63, 31.25, and 62.50 *μ*g/mL BNC-pretreated groups decreased with increasing concentration of BNC (Figure 4).

### 3.5. BNC Protected H9c2 Cells from Damage Caused by H_**2**_O_**2**_-Induced Oxidative Stress

**Figure 5:**
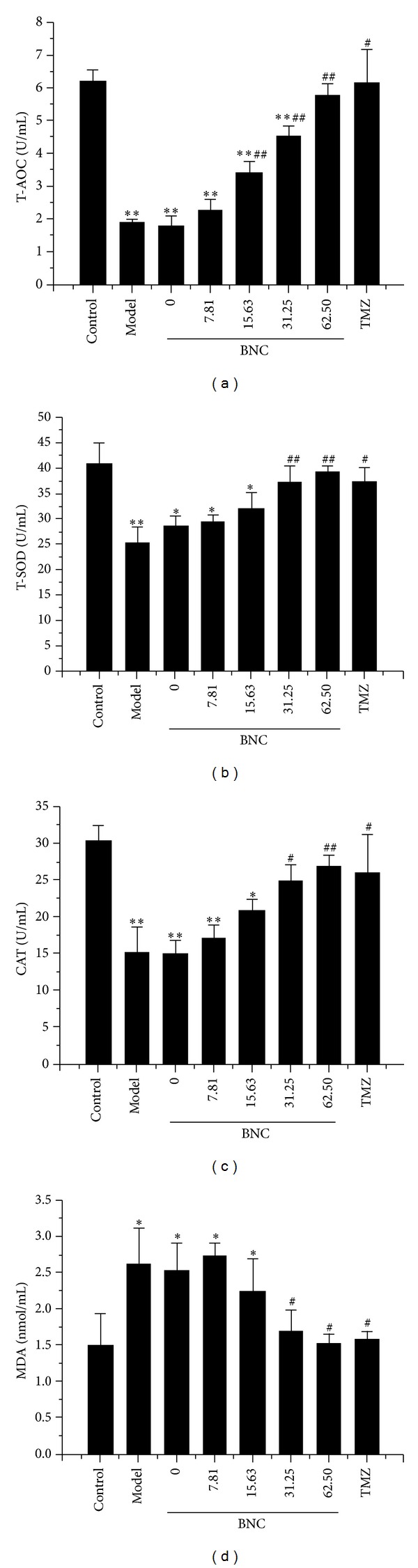
Protective effects of BNC via enhancing antioxidant function by increasing the activities of T-AOC, T-SOD, and CAT and decreasing the production of MDA detected by the T-AOC, T-SOD, CAT, and MDA assay kits. Values are expressed as mean ± SD from three independent experiments. **P < 0.01 versus control; *P < 0.05 versus control; ^##^ *P* < 0.01 versus model; ^#^ *P* < 0.05 versus model.

Oxidative stress has been implicated in the pathogenesis of myocardial injury. To determine whether BNC affects oxidative stress-related biochemical enzymes, the levels of oxidant and antioxidant enzymes, such as T-AOC, T-SOD, CAT, and MDA, were measured in H9c2 cell lysates. The activities of T-AOC, T-SOD, and CAT were increased in a dose-dependent manner in the BNC pretreatment group relative to the H_2_O_2_ group, whereas MDA production was reduced (Figure 5). These results confirmed that the in vitro antioxidant capacity of BNC was comparable to that of TMZ.

### 3.6. BNC Attenuated Intracellular ROS Generation

**Figure 6:**
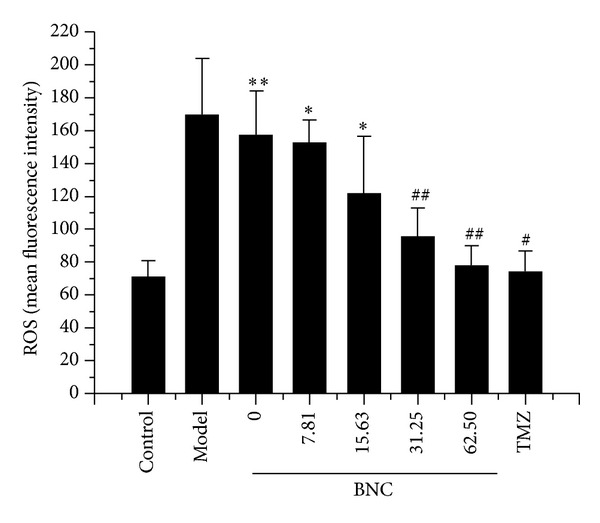
Protective effects of BNC due to reduced H_2_O_2_-induced generation of intracellular ROS measured by the DCFDA assay. Values are expressed as mean ± SD from three independent experiments. **P < 0.01 versus control; *P < 0.05 versus control; ^##^ *P* < 0.01 versus model; ^#^ *P* < 0.05 versus model.

The generation of intracellular ROS by H_2_O_2_ promotes cellular damage. Intracellular ROS concentrations were measured with the DCFH-DA assay to determine if BNC attenuated cell death by reducing ROS generation. Intracellular ROS generation was significantly increased in H_2_O_2_-treated H9c2 cells. However, ROS generation was significantly reduced by BNC pretreatment in a concentration-dependent manner (Figure 6).

### 3.7. BNC Protected H9c2 Cells from H_**2**_O_**2**_-Induced Apoptosis

**Figure 7:**
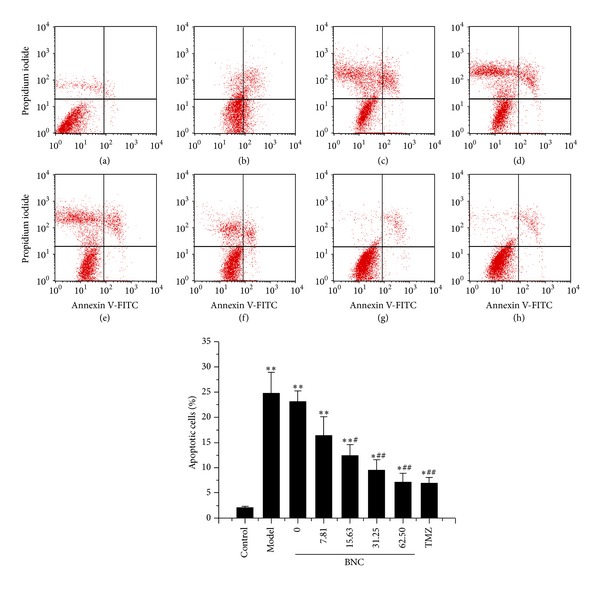
BNC protected H9c2 cells from H_2_O_2_-induced apoptosis, as detected by Annexin V-FITC and PI staining with FCM analysis. (a) H9c2 cells without BNC or H_2_O_2_ (control); (b) H9c2 cells exposed to 100 *μ*M H_2_O_2_ (model); ((c)–(h)) H9c2 cells pretreated with 0, 7.81, 15.63, 31.25, 62.50 *μ*g/mL BNC, or 10 *μ*M TMZ followed by the treatment of 100 *μ*M H_2_O_2_. Values are expressed as mean ± SD from three independent experiments. **P < 0.01 versus control; *P < 0.05 versus control; ^##^ *P* < 0.01 versus model; ^#^ *P* < 0.05 versus model.

The percentage of apoptotic cells was determined by Annexin V-FITC and PI staining to quantify the effects of BNC on H_2_O_2_-induced H9c2 apoptosis. The numbers of apoptotic cells were significantly increased upon treatment with 100 *μ*M H_2_O_2_ in comparison to the untreated control group (24.54 ± 5.27% versus 2.05 ± 0.12%, *P* < 0.01). Cotreatment of H9c2 cells with 100 *μ*M H_2_O_2_ and BNC (7.81, 15.63, 31.25, or 62.50 *μ*g/mL) for 24 h reduced the percentages of apoptotic cells to 16.56 ± 3.55%, 12.81 ± 2.64%, 9.48 ± 2.19%, and 7.18 ± 1.97%, respectively. The survival rates for the blank control group and the TMZ group were 23.19 ± 4.28% and 6.49 ± 1.32%, respectively (Figure 7). These experiments suggested that BNC protected cardiomyocytes by decreasing H_2_O_2_-induced early apoptosis. It also showed that BNC was equivalent to TMZ in protecting H9c2 cells from H_2_O_2_-induced apoptosis.

### 3.8. BNC Decreased Cytosolic Ca^2+^ Concentrations

**Figure 8:**
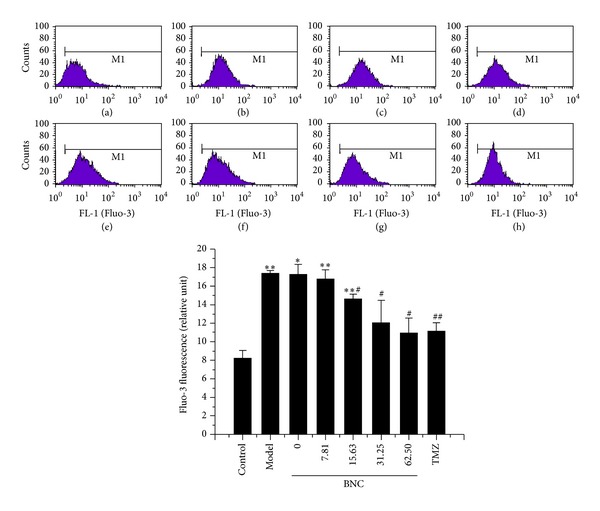
BNC reduced H_2_O_2_-induced cytosolic Ca^2+^ concentrations in H9c2 cells, as observed by Fluo-3/AM staining with FCM analysis. (a) H9c2 cells without BNC or H_2_O_2_ (control); (b) H9c2 cells exposed to 100 *μ*M H_2_O_2_ (model); ((c)–(h)) H9c2 cells pretreated with 0, 7.81, 15.63, 31.25, 62.50 *μ*g/mL BNC, or 10 *μ*M TMZ followed by the treatment with 100 *μ*M H_2_O_2_. Values are expressed as mean ± SD from three independent experiments. **P < 0.01 versus control; *P < 0.05 versus control; ^##^ *P* < 0.01 versus model; ^#^ *P* < 0.05 versus model.

Intracellular Ca^2+^ accumulation and destruction of Ca^2+^ homeostasis are thought to initiate myocardial injury. Cytosolic Ca^2+^ concentrations were significantly higher in H_2_O_2_-treated H9c2 cells than in control cells, as measured by Fluo-3/AM staining. Furthermore, the cytosolic Ca^2+^ concentrations in H_2_O_2_-treated H9c2 cells pretreated with BNC (7.81, 15.63, 31.25, or 62.50 *μ*g/mL) were reduced in a dose-dependent manner (Figure 8). 

### 3.9. BNC Rescued Loss of Mitochondrial Membrane Potential in H_**2**_O_**2**_-Treated H9c2 Cells

**Figure 9:**
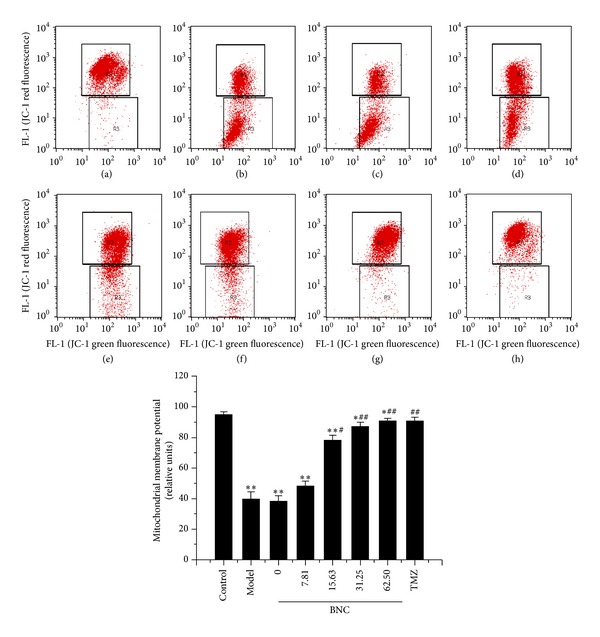
BNC attenuated H_2_O_2_-induced mitochondrial membrane potential loss in H9c2 cells, as assessed by JC-1 staining with FCM analysis. (a) H9c2 cells without BNC or H_2_O_2_ (control); (b) H9c2 cells exposed to 100 *μ*M H_2_O_2_ (model); ((c)–(h)) H9c2 cells pretreated with 0, 7.81, 15.63, 31.25, 62.50 *μ*g/mL BNC or 10 *μ*M TMZ followed by treatment of 100 *μ*M H_2_O_2_. Values are expressed as mean ± SD from three independent experiments. **P < 0.01 versus control; *P < 0.05 versus control; ^##^ *P* < 0.01 versus model; ^#^ *P* < 0.05 versus model.

The loss of mitochondrial membrane potential is an important event of the apoptotic process. The JC-1 assay was used to determine whether mitochondria participated in H_2_O_2_-induced apoptosis. The mitochondrial membrane potential was markedly decreased in H9c2 cells treated with 100 *μ*M H_2_O_2_ for 1 h, indicating that H_2_O_2_ treatment induced mitochondrial dysfunction (Figure 9). BNC pretreatment significantly reduced the loss of H_2_O_2_-induced mitochondrial membrane potential in a dose-dependent manner.

### 3.10. BNC Altered PARP and Caspase-3 Expression

**Figure 10:**
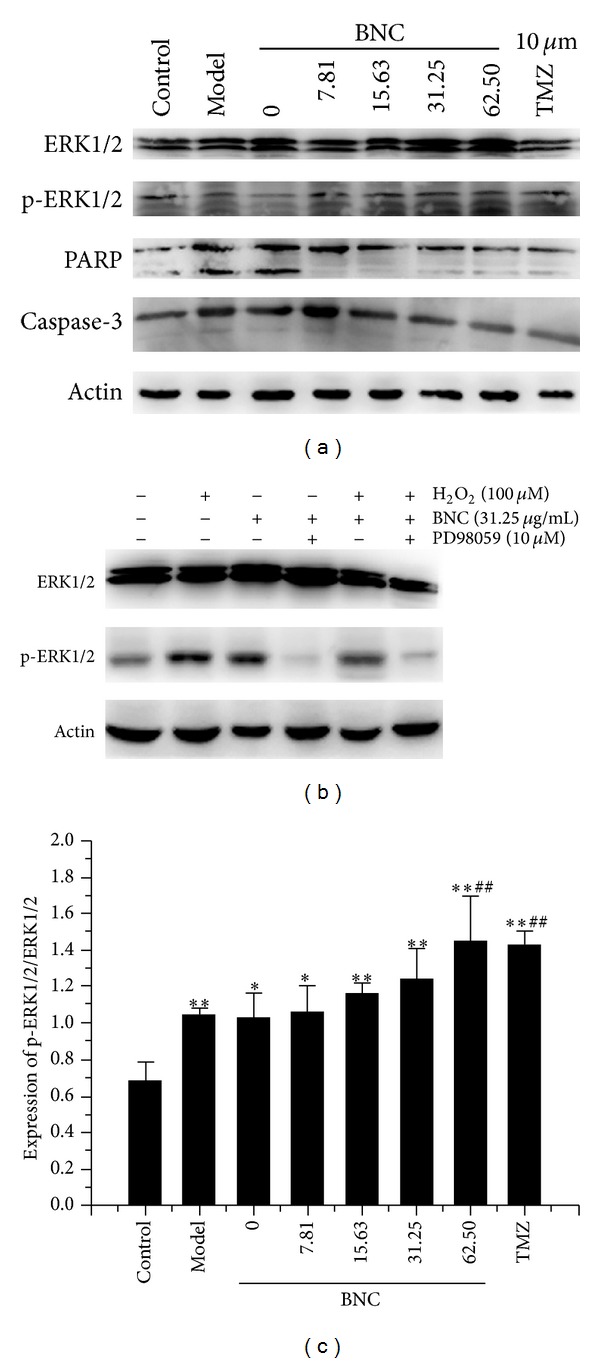
Effects of BNC on expression of ERK1/2, p-ERK1/2, PARP, and caspase-3 in H9c2 cells treated with H_2_O_2_ (a) and effects of BNC on expression of ERK1/2 and p-ERK1/2 in the presence of ERK1/2 inhibitor, PD98059 (10 *μ*M) (b). Protein expression was detected by western blot analysis. The ratio of p-ERK1/2/ERK1/2 significantly increased in response to BNC pretreatment (c). Values are expressed as mean ± SD from three independent experiments. **P < 0.01 versus control; *P < 0.05 versus control; ^##^ *P* < 0.01 versus model.

PARP and caspase-3 activation are important biomarkers in the apoptotic process. Protein levels were examined by western blot analysis to investigate whether BNC protects H9c2 cells from H_2_O_2_-induced apoptosis. Caspase-3 activity was significantly increased in H9c2 cells treated with 100 *μ*M H_2_O_2_ in comparison to control cells. Co-treatment of H9c2 cells with 100 *μ*M H_2_O_2_ and BNC (7.81, 15.63, 31.25, or 62.50 *μ*g/mL) for 24 h significantly suppressed caspase-3 activity in a dose-dependent manner in comparison to H_2_O_2_-treated cells. Changes in PARP were also significantly altered. PARP cleavage was clearly detected in the H_2_O_2_ group, whereas PARP cleavage was not observed in the BNC group (Figure 10(a)). These results showed that BNC (7.81, 15.63, 31.25, or 62.50 *μ*g/mL) prevented H_2_O_2_-induced apoptosis.

### 3.11. BNC Induced Early Phosphorylation of ERK1/2

Mitogen-activated protein kinase (MAPK) family members were investigated to be involved in BNC-stimulated signaling. Protein lysates were collected and examined by western blot analysis for phospho-ERK1/2 and ERK1/2. Phosphorylation of ERK1/2 was moderately elevated after 1 h of H_2_O_2_ treatment (Figure 10(a)). However, pretreatment with BNC induced phosphorylation of ERK1/2 more rapidly, with increased band intensity present at 1 h. Expression of p-ERK1/2/ERK1/2 was significantly increased in cells preconditioned with BNC in comparison to the H_2_O_2_ group (Figure 10(c)). Pretreatment of cells with the highly selective ERK1/2 inhibitor, PD98059 (10 *μ*M), indicated that ERK1/2 inhibition blocked the protective effect of BNC in H_2_O_2_-treated cells (Figure 10(b)).

## 4. Discussion

Oxidative stress in cardiomyocytes has an important role in the pathogenesis of many cardiovascular diseases, and ROS generated by oxidative stress are central to cardiac injury [17]. Studies have shown that mitochondria play prominent roles in the transduction and amplification of the apoptotic response in cardiomyocytes during oxidative stress [18, 19]. ROS promote the release of cytochrome c by increasing mitochondrial permeability. Pro-caspase-3 is downstream of cytochrome c in the apoptotic cascade and is cleaved by active caspase-9 to produce active caspase-3. In addition to caspases-6 and caspases-7, caspase-3 contributes to oligonucleosomal DNA fragmentation, which ultimately leads to apoptosis [20, 21]. Ca^2+^ is critical for cardiomyocyte contractility and for the signaling pathways in cardiac growth and remodeling. Cytosolic Ca^2+^ increases abnormally in cardiomyocytes as a consequence of ischemic reperfusion injury or other stresses, causing myocardial dysfunction and cell death. Increased Ca^2+^ activates calpains, leading to disruption of plasma membrane. Calpain activates the proapoptotic protein, BID. Calpain also cleaves autophagy protein 5, shifting the balance from autophagy to apoptosis [22]. We found that BNC inhibited H_2_O_2_-induced activation of PARP and caspase-3, reduced intracellular Ca^2+^ concentrations, and improved H_2_O_2_-induced impairment of the mitochondrial membrane potential. The present study clearly demonstrated that BNC inhibited H_2_O_2_-induced apoptosis by blocking Ca^2+^-dependent and mitochondria- dependent apoptosis.

The balance between ROS production and clearance of endogenous antioxidants is destroyed during oxidative stress, resulting in upregulation of endogenous antioxidants. Major endogenous antioxidants present in cardiomyocytes include SOD, CAT, GSH, glutathione peroxidase, ubiquinone, and vitamins. Our results suggested that BNC protected H_2_O_2_-induced injury by enhancing antioxidant abilities, such as increasing T-AOC, T-SOD, and CAT activities and decreasing ROS and MDA content. 

**Figure 11:**
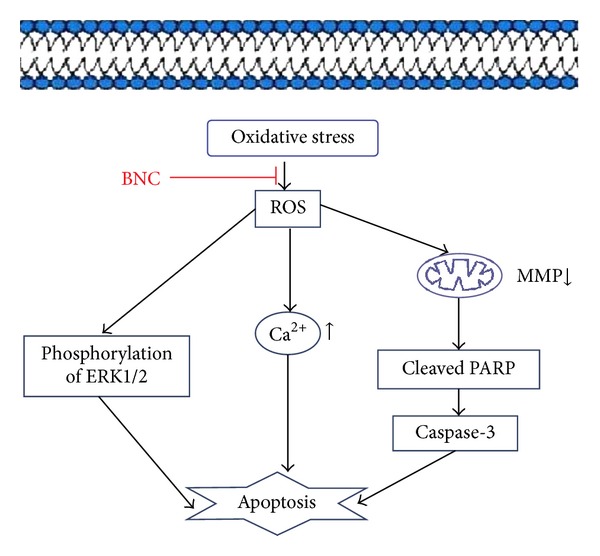
Schematic representation of BNC-mediated protection from H_2_O_2_-induced oxidative injury in H9c2 cells.

MAPKs are serine/threonine protein kinases involved in cellular proliferation, differentiation, and death [23, 24]. Activation of the ERK1/2 MAPK subfamily in cardiac myocytes regulates expression of many genes and relates to the development of cellular hypertrophy. Activation of ERK1/2 in response to H_2_O_2_ is mediated through the Ras/Raf1/Mek pathway [25]. ERK1/2 activation may promote inflammation and result in necrosis by up-regulating interleukin-1*β* [26]. ERK1/2 activation may also inhibit apoptosis by down regulating Bad and up-regulating Bcl-2 [27]. Our research indicated that BNC exerted a protective effect against oxidative stress-mediated injury in H9c2 cells by inducing early phosphorylation of ERK1/2 (Figure 11).

BNC is a traditional Chinese medicine for the treatment of cerebrovascular and cardiovascular diseases. BNC intestinal absorption liquid was used in this study instead of drug-containing serum and extraction solution. Traditional Chinese medicines are usually consumed orally. Thus, the small intestine is a major absorption site for herbal medicines. However, herbal mixtures contain multiple ingredients, and complicated herb-herb interactions can occur during intestinal absorption [28]. The process for producing intestinal absorption liquids imitates the complicated absorption process of traditional Chinese medicine. Absorbed constituents from the digestive tract may produce therapeutic effects [29]. UPLC analysis further confirmed that the compounds in BNC intestinal absorption liquids are similar to the compounds present in BNC extraction solution. Our studies lay a foundation for illuminating the cardioprotective effect of BNC preconditioning on oxidative stress damage. However, further studies need to be performed to understand and develop new strategies for cardioprotection during oxidative stress.

TMZ is a clinically effective antianginal drug that has direct cytoprotective effects on the myocardium. TMZ reduces oxygen free radical production at the cellular level [30], reduces intracellular calcium overload, and improves mitochondrial metabolism [31]. Mesenchymal stem cells that were preconditioned with TMZ before implantation significantly enhanced the recovery of myocardial function by increasing gene and protein expression levels of HIF-1, survivin, p-Akt, and Bcl-2. Our data demonstrated that H_2_O_2_-induced impairment was attenuated in TMZ-preconditioned H9c2 cells. These results are consistent with previous studies. These data indicated that the potency of BNC for protecting H9c2 cells from oxidative damage is similar to that of the positive control, TMZ.

## 5. Conclusion

BNC protected H9c2 cardiomyocytes from H_2_O_2_-induced oxidative injury by enhancing antioxidant abilities, activating ERK1/2 signaling, inhibiting apoptosis-related signal transduction pathways, reducing intracellular Ca^2+^ concentrations, and improving mitochondrial membrane potential. This research provides evidence that BNC exerted a protective effect on cardiovascular damage and the potency of BNC for protecting H9c2 cells from oxidative injury is comparable to that of trimetazidine.
